# Gangrene of the Lungs

**Published:** 1859-09

**Authors:** E. J. Fountain

**Affiliations:** Davenport, Iowa


ART. II.—*Gangrene of the Lung, resulting from a Foreign Substance lodged in the Right Bronchial Tube, and terminating in Empyema, and Perforation through the Diaphragm and into the Colon. Final Expulsion of the Body and Recovery of the Patient.* By E. J. FOUNTAIN, M.D., of Davenport, Iowa.

THE following case presents a series of phenomena of a very rare, if not unprecedented occurrence, resulting from a foreign body in the lungs. I

was called on the 29th of November, 1858, to see the patient, Alexander Barrow, an interesting lad, ten years of age, whose health had been generally very good, though of a delicate constitution. I found him laboring under a severe cough, of a dry, hoarse, and resonant character, occurring in frequent paroxysms, and unattended with any expectoration. On placing the patient in an upright position, to remove his garments, for the purpose of examining his chest, symptoms of fainting appeared, requiring an immediate resort to the recumbent position. This induced me to remark that his difficulty must be of a serious character, but I could not as yet even guess at its nature. I proceeded carefully to examine his chest, the first result of which increased the mystery of his case. Everywhere over the left lung the respiratory murmur was clear and distinct, and entirely free from the slightest crepitation or the least symptom of disorder. The upper lobe of the right lung appeared equally healthy, but in the middle and lower part there was an entire absence of all respiratory sounds. There was an appreciable degree of dullness over all this region, but more marked anteriorly, and pain was felt on percussion at a single point near the margin of the fourth rib, a little to the right of the sternum. I now inquired more particularly into the history of the cough, and learned that it had existed since he was suddenly taken with a violent paroxysm of coughing at the dinner-table, on the nineteenth of the same month, ten days before coming under my observation. This gave me a clue as to the nature of his difficulty, and I inquired very particularly concerning the circumstances relating to this occurrence. The patient stated that he had bitten off the round head of the thigh-bone of a chicken, and at the moment of doing this he became strangled by some crumbs of corn-bread which he also had in his mouth at the same time, and with the first effort of coughing the bone was drawn into his throat. This was followed by a long and violent paroxysm of coughing; during the remainder of the day it continued most of the time, and in the intervals his breathing was accompanied with a hoarse, croupy sound. The cough had continued unremittingly from that period, yet, very singularly, it had excited no suspicion in the minds of his parents of the probability or danger of the bone having entered the trachea. The suddenness of the attack, with the circumstances related, taken in connection with the symptoms revealed by auscultation, immediately impressed my mind with the belief that his cough was caused by the articulating head of the thigh-bone of a chicken lodged in the right bronchial tube, immediately beyond the first branch which is given off to supply the upper lobe of the lung. On the day following I examined his chest very carefully with the aid of Cammann's double stethoscope, the result of which more than confirmed the opinion which I had entertained the evening previous. As before, I found the left lung everywhere in a perfectly healthy and normal condition, but the respiration puerile. The respiratory murmur was perfectly clear in the upper part of the right lung, but on passing downward with the stethoscope, I found that all sounds suddenly ceased about the fourth rib. Above this line the voice could be heard through the stethoscope as from a healthy lung; below, it was altogether wanting, the voice reaching the ear only from without. There was some degree of dullness also below this line, and at a point corresponding to the root of the lung there was pain on percussion. From these symptoms I concluded that some foreign body had lodged in the right bronchial tube, between its first and second bifurcation, and it was further evident to my mind that the substance must be something which was of such a shape as to completely fill the calibre of the tube. The round and polished head of a chicken leg-bone would, by its size and form, exactly accomplish this, and the history of the origin of the cough rendered it in the highest degree probable that this constituted the obstruction, and was the cause of all his trouble. To the parents I stated this as my unqualified opinion, and expressed my apprehension that it would be a serious case, unless in some way the bone could be expelled by coughing, or otherwise removed. The operation of tracheotomy I thought would be of no benefit, as the substance was fixed in a position too far to be reached by any instrument with which extraction might be attempted with any prospect of success. Moreover, my diagnosis of the case was received with so much incredulity that I felt certain that any proposition of an operation would have been immediately opposed by the family and their friends. I concluded it would be best to trust almost entirely to nature, with the hope that it would be expelled by coughing, after the bone should become loosened from its position by partial decomposition of its substance and suppuration of the parts surrounding it. I prescribed an expectorant and tonic mixture, containing iodide of potassium and the compound tincture of bark, to promote expectoration, and thereby favor the loosening and expulsion of the bone. To facilitate this, I also directed the patient to rest occasionally, during the paroxysms of coughing, with his arms and face upon the carpet, while his body remained upon a low couch. The directions were repeatedly observed, but no benefit resulted from the attempts to favor the removal of the body by aid of gravity. After a few days he began to expectorate a little, and evident signs of improvement followed. By examination of the chest, it appeared that the increased bronchial secretion had loosened the bone a little from its previously firm position, as a feeble current of air could be heard to pass by the point of obstruction. This improvement in his symptoms encouraged in my mind the hope that, after partial decomposition of the bone by absorption, the solid nucleus of osseous matter would be expelled by an effort of coughing. The expectorant and tonic treatment was continued unchanged. For about three weeks he slowly improved in strength, and regained his natural bouyancy of spirits; but still the cough remained persistent and unchanged in character. During this time I called in, at different times, Drs. Adler and Baker, both of whom agreed with me fully in my opinion of the case, and also in the propriety of trusting to the chances of a natural expulsion of the foreign body from the place in which it was lodged. By the twenty-eighth of December his symptoms became more unfavorable. Loss of animation and general strength was soon followed by complaints of pain in the hip, as he expressed it, leading his parents to think he was suffering from a rheumatic difficulty. On visiting the patient the day following, I found him very much altered in appearance, which was expressive of great suffering.

The pain which he referred to the hip proceeded from a diffused tumefaction over the lower border of the ribs. The tenderness over this region was so great that he could not tolerate a thorough examination. The slightest touch would provoke resistance and cries of pain. His breath was fetid, and the cough continued of the same character, only the paroxysms were more frequent. The symptoms revealed by auscultation were unchanged, except an increased amount of dullness and tenderness over the submaxillary region. I gave it as my opinion that an extensive abscess had formed in the lung, resulting from a gangrenous condition of the parts below the point of obstruction, and that the matter was emerging from the chest in an unusual way, by passing through the attachments of the diaphragm, and forming a tumor over the border of the ribs, simulating in appearance an abscess of the liver. The pain increased in severity as the swelling advanced. The patient rested in a semi-recumbent position, with the thighs strongly flexed upon the body, by which the parietes of the abdomen were relaxed, and the pressure over the tumor diminished.

In consultation with my friends at this time, it became a question whether this abscess was from the liver or the lungs, and at times some doubt was entertained as to the source of the tumor. The patient's strength failed rapidly. The fetor of his breath was arrested by the administration of the chlorate of potassa, and the bark and iodide of potassium were prescribed as an alterative and tonic.

January 1st. The tumor now increasing antero-posteriorly, and presenting no appearance of pointing. Pain and tenderness over the whole of the right side below the fourth rib. These facts confirmed me in the opinion that the tumor was formed by purulent effusion within the chest. Prognosis very unfavorable. On the night following I was sent for in haste, and found him sinking rapidly, and in the most extreme agony of pain. An expression of intense suffering escaped with almost every breath, and the slightest effort to change his position was always attended with such cries of agony as to unnerve the stoutest heart. To his parents the trial was peculiarly severe, and called forth from his friends the warmest sympathy and the most devoted attendance. At this time his suffering was partially relieved by opiates, but during the night his condition appeared extremely critical, and hardly any expectation was entertained of his recovery. On the day following Dr. Grave was called in consultation. By him the difficulty was regarded as an abscess of the liver, and he appeared altogether incredulous of the existence of any foreign substance in the lungs. Fomentations were applied to the side very assiduously, to the great annoyance of the patient, as the slightest pressure upon the side or movement of the body occasioned immediate and severe pain. This was considered necessary, to favor the pointing of the supposed hepatic abscess. Internally the consulting physician prescribed cherry-laurel water, and directed the use of opiates to be discontinued. For a time my opinions and advice were disregarded, and the case was treated as an abscess of the liver, while the constant cough and peculiar symptoms of disorder of the lungs were quite overlooked, or considered unimportant. As the influence of the opium passed off, his suffering returned as severe as before, and for several days and nights he had no respite from pain of the most agonizing character. Opiates were now gladly resorted to again, and followed by immediate relief. He was allowed to return to the use of wine and bark, and on the seventh I added the compound syrup of the phosphates. During this time he was becoming more emaciated, and it was apparent that he was gradually failing. No pointing of the tumor had yet appeared; on the contrary, it extended transversely across the body, along the lower border of the ribs, reaching at this time nearly to the spine, and measuring ten inches in its antero-posterior diameter. No fluctuation could yet be detected, but on the tenth an exploring operation was decided upon, and the day following, assisted by Drs. Adler and Grave, I introduced an exploring needle, along the groove of which a drop of pus immediately appeared. Withdrawing this, I introduced a small trocar and canula, and drew off nearly a pint of extremely offensive pus. The odor was peculiarly sickening and pungent, and identical with that which I have noticed proceeding from gangrene of the lung, when revealed by post-mortem examination, only of far greater intensity

Fearing there might not be an adhesion between the peritoneal covering of the tumor and that of the abdominal parietes, I opened the tumor by a puncture merely; and, after the evacuation of the pus, I closed the wound tightly by adhesive plaster. By the time the pus should reaccumulate there would necessarily be an adhesion about the point of puncture, through which a free opening could be made without danger of allowing the pus to escape into the cavity of the peritoneum, provided an operation should be again necessary; for I entertained the opinion that it might soon find a natural exit from the body by ulceration into the colon, upon the angle of which I thought the tumor to be impinging. The extensive tumefaction had subsided only in part by the discharge, after which it began to enlarge again in the same form as before. We decided to operate again on the fourteenth, but on the morning of this day he suddenly began to pass by the bowels pus of precisely the same odor and character as that removed from the tumor. Five or six evacuations followed in rapid succession, each one filling the house with the same extremely offensive odor. As these evacuations were going on the tumor subsided, and the patient became greatly relieved of his previous suffering. It was at once evident that the sac had opened into the colon; and as this would be at the deepest and most dependent part of the tumor, we believed it would permit a more complete and constant escape of the pus than by an external opening; and from this consideration I was disposed to regard the occurrence as favorable, and affording the patient a little better chance of recovery. In the mean time the cough continued unremittingly the same, and auscultation still revealed plainly enough to my mind the fact that some foreign body still obstructed the respiration in the right lung. As the discharges continued, the pain gradually subsided, and soon a slow and gradual improvement was apparent. The discharges of pus by the bowels continued about a week, each evacuation being preceded by a sharp pain in the region of the arch of the colon on the right side. About this time the patient had a more than usually violent paroxysm of coughing; after which he appeared much relieved, and the cough almost entirely ceased. On examining the chest, I found the air entered more freely into the lung than previously; and these facts led me to infer that the bone had either been loosened in its position, or it had been coughed up and swallowed. The tumor first subsided over the lateral region, leaving an elevation posteriorly over the lower part of the chest, extending nearly to the spine, and mounting upward over the ribs. This convinced me of the correctness of my diagnosis as to the cause and source of the abscess, and it was farther confirmed by the facility with which the fingers could be inserted under the ribs, and pressed, without producing any pain, upon the lower margin of the liver. There was the same dullness over all the inferior portion of the chest, and some tenderness on percussion. The discharges of pus gradually ceased, but the cough soon returned again as before. In the mean time the patient steadily improved in strength, and apparently was rapidly recovering. As soon as he was able to travel, the family left for the East early in February, by which time the patient could walk about the house without pain, and with considerable freedom of motion. I was still as positive as ever that all his symptoms resulted from a bone in the lungs, and expressed the opinion that, if still remaining, it would probably be in part absorbed, and the remainder might yet be coughed up at some future time.

The remaining history I will give by extracts from letters written to me by Mr. Barrow, from Baltimore. The first was dated March 3d, 1859, of which the following is a portion:—

"Believing that you will be interested in the progress of Alexander's case, I hasten to inform you that your diagnosis of his disease has proven correct. *He has thrown up the bone.* The substance when discharged was about the size of a common garden pea, and *solid bone*, and was undoubtedly a part of a chicken-leg bone. It was very much decomposed, but in its present dry state still shows the grain of the bone. After he left Davenport, he improved rapidly in strength, and fattened up very much, but his cough still continued. He has *not coughed at all* since the bone was expelled, and I now have hope of his ultimate recovery." At the time of writing this he was having symptoms of intermittent fever.

On the ninth of April I received from Mr. Barrow another letter, inclosing the bone, "or rather," he writes, "the little that is left of it; it having been much broken and wasted by handling. After I wrote you, Alexander continued quite sick for four or five days, with high fever and some inflammation of the right lung. He is now rapidly recovering, and seems quite sound, not a vestige of cough remaining."

On the nineteenth of May, he writes again as follows: "Since the last attack, of which I wrote you in a former letter, Alexander has been steadily growing in health and strength. He is, of course, still quite weak in that lung, and unable to engage in violent plays, but there is every prospect of his becoming a stout and hearty boy."

In relation to the diagnosis of this case, it will be observed that I was supported by two practitioners, who were called in consultation, and opposed by another, who, for a time, had almost entire charge of the patient. I cannot better express the embarrassment to which I was subjected than by the following quotation from the same letter last referred to:—

"In the matter of Alexander's sickness," he writes, "we were well aware, at the time, of the doubts, and, in some cases, sneers, expressed by our mutual acquaintances, as to your diagnosis of Alexander's disease; which more than once, I am free to confess, somewhat shook my confidence, not in your skill as a physician, but in your correctness in that particular case; and we had frequently to listen to learned arguments to prove the impossibility of such a state of things as you claimed existed. Even Dr. Grave himself gravely doubted the existence of the bone in his lungs. This doubt was also *very decidedly* expressed by two skillful physicians of Baltimore, acquaintances of mine, to whom I stated the case. Under all the circumstances, I declare to you, that the subsequent *proof* gave me perhaps as much pleasure as it did you; for it was a convincing proof that our friends, in and out of the profession, had not done you justice."

In Dr. Gross's work on "Foreign Bodies in the Air-Passages," I find no case like this in the remarkable phenomena which resulted from the introduction of a foreign substance into the lungs. In a number of cases, abscesses were found in the lung, and a quantity of pus thrown up along with the foreign body; but in none did the matter take such a peculiar course as in the case I have just related.

In some of the cases which terminated fatally, a large accumulation of pus was found in the cavity of the chest, and in a few the abscesses opened externally through the intercostal spaces; but in no instance had the ulceration extended through the diaphragm into the intestinal canal. The nearest approach to this case is one related by Dr. Watson, furnished him by Mr. Herbert Mayo, of London. "The foreign substance was an ear of rye, which occasioned symptoms of pulmonary irritation, attended with hectic fever, and with the most fetid expectoration. On post-mortem examination, the ear of rye was found in an abscess which was common to the right lung and to the liver, the latter of which had become involved by an extension of the morbid action across the diaphragm."

In many cases of obstruction of the air-passages the chest is unnaturally clear and resonant. In this case the dullness corroborates the statement of Dr. Gross, as necessarily resulting from the complete obstruction of the passage by the form and size of the bone, which not only prevented the entrance of air, but also the evaporation and discharge of the bronchial secretions.

It may be well to consider whether in this case the operation of tracheotomy should have been performed, and an attempt made to remove the bone. From the history it will be apparent that it was impracticable, from the disposition to discredit the opinion which I entertained of the nature of the difficulty; but I doubted then, and still entertain a doubt, of its propriety in this instance, on account of the form of the substance, its close impaction, and the depth to which it had descended in the lung. Had the same substance lodged in the larynx, trachea, or primary bronchial tube, of course an operation should have been immediately resorted to; and even if lodged in the second division of the bronchial tube, as in this case, an operation might be performed with some chance of success, provided the substance had an irregular form or uneven surface. Even where the substance has been lodged just below the bifurcation of the trachea, the operation has been performed by as skillful a surgeon as the late Dr. McClellan, of Philadelphia, aided by Drs. Gross and Sharp, and repeated attempts made to extract the foreign body, a kidney bean, without success. In this instance, the patient died from the effects of the ensuing inflammation of the lung, nine days after the operation. The result in this case might reasonably enough discourage a similar attempt in one so much more unfavorable as the subject of this report.

After reflecting upon the history of the cases reported by Dr. Gross, I am impressed with the belief that my original opinion was correct in deciding against the practicability of a successful operation, although, by the directions given by this eminent author, I would have been justified in making the attempt. I think it would have been unsuccessful had it been attempted; and it might have excited an inflammation in the upper part of the lung, or otherwise aggravated the disorder in the chest to such a degree as to occasion a fatal termination, and thus sacrifice a life which, perhaps, escaped such a fate by trusting in the prospect of spontaneous expulsion.

